# Renal impairment after switching from stavudine/lamivudine to tenofovir/lamivudine in NNRTI-based antiretroviral regimens

**DOI:** 10.1186/1742-6405-7-37

**Published:** 2010-10-11

**Authors:** Weerawat Manosuthi, Wiroj Mankatitham, Aroon Lueangniyomkul, Wisit Prasithsirikul, Preecha Tantanathip, Busakorn Suntisuklappon, Anongnuch Narkksoksung, Samruay Nilkamhang, Somnuek Sungkanuparph

**Affiliations:** 1 Bamrasnaradura Infectious Diseases Institute, Ministry of Public Health, Nonthaburi, Thailand; 2 Faculty of Medicine Ramathibodi Hospital, Mahidol University, Bangkok, Thailand

## Abstract

**Background:**

During stavudine phase-out plan in developing countries, tenofovir is used to substitute stavudine. However, knowledge regarding whether there is any difference of the frequency of renal injury between tenofovir/lamivudine/efavirenz and tenofovir/lamivudine/nevirapine is lacking.

**Methods:**

This prospective study was conducted among HIV-infected patients who were switched NRTI from stavudine/lamivudine to tenofovir/lamivudine in efavirenz-based (EFV group) and nevirapine-based regimen (NVP group) after two years of an ongoing randomized trial. All patients were assessed for serum phosphorus, uric acid, creatinine, estimated glomerular filtration rate (eGFR), and urinalysis at time of switching, 12 and 24 weeks.

**Results:**

Of 62 patients, 28 were in EFV group and 34 were in NVP group. Baseline characteristics and eGFR were not different between two groups. At 12 weeks, comparing mean ± SD measures between EFV group and NVP group were: phosphorus of 3.16 ± 0.53 vs. 2.81 ± 0.42 mg/dL (*P *= 0.005), %patients with proteinuria were 15% vs. 38% (*P *= 0.050). At 24 weeks, mean ± SD phosphorus and median (IQR) eGFR between the corresponding groups were 3.26 ± 0.78 vs. 2.84 ± 0.47 mg/dL (*P *= 0.011) and 110 (99-121) vs. 98 (83-112) mL/min (*P *= 0.008). In NVP group, comparing week 12 to time of switching, there was a decrement of phosphorus (*P *= 0.007) and eGFR (*P *= 0.034). By multivariate analysis, 'receiving nevirapine', 'old age' and 'low baseline serum phosphorus' were associated with hypophosphatemia at 24 weeks (*P *< 0.05). Receiving nevirapine and low baseline eGFR were associated with lower eGFR at 24 weeks (*P *< 0.05).

**Conclusion:**

The frequency of tenofovir-associated renal impairment was higher in patients receiving tenofovir/lamivudine/nevirapine compared to tenofovir/lamivudine/efavirenz. Further studies regarding patho-physiology are warranted.

## Introduction

The therapeutic goal of antiretroviral therapy (ART) in human immunodeficiency virus (HIV)-infected patients is to maintain undetectable plasma HIV viral load and reduce HIV-associated morbidity and mortality. However, long-term exposure to ART may also be associated with its significant toxicity [1]. Tenofovir is a nucleotide reverse transcriptase inhibitor with potent activity against HIV. According to the current HIV treatment guidelines, tenofovir is one of the drugs recommended use in the initial backbone for first-line HIV treatment [1,2]. This drug generally has few side effects or toxicities; the most common adverse events identified from the large controlled clinical trials include skin rashes, nausea, flatulence, diarrhea, and headache [3,4]. Tenofovir is principally eliminated via the kidney; nevertheless, minimal reductions in renal function have been reported in the patients treated with tenofovir [5]. Severe renal toxicity, including acute renal failure and Fanconi syndrome, has been reported infrequently so far [3,4,6-8]. However, it is recommended that creatinine clearance should be calculated prior to initiating this drug as well as routine monitoring of creatinine clearance and serum phosphorus should be performed [1,9].

On the other hand, a generic fixed dose combination of stavudine, lamivudine, and nevirapine has been widely prescribed in resource-constrained countries until recently [10]. Given that progressive reduction in the use of stavudine is undertaken due to stavudine-related toxicities, either tenofovir or zidovudine has been used to substitute during stavudine phase-out plan in many developing countries. Most of what the previous studies show regarding tenofovir-related toxicities was studied in a regimen of efavirenz-based or protease inhibitors-based ART and data of tenofovir-containing backbone NRTI plus nevirapine is scanty [11,12]. Knowledge regarding whether there is any difference of the frequency of renal injury after switching stavudine to tenofovir between a regimen of tenofovir, lamivudine, and efavirenz versus tenofovir, lamivudine, and nevirapine is still lacking.

## Methods

The N2R study was a prospective, open-label, randomized trial involving 142 adult Thai patients (71 patients per group) co-infected with HIV and TB to study two NNRTI-based ART, included efavirenz-based (EFV group) and nevirapine-based regimen (NVP group) at Bamrasnaradura Infectious Diseases Institute, Ministry of Public Health, Nonthaburi, Thailand [13]. The initial nucleoside reverse transcriptase inhibitor (NRTI) backbone was stavudine and lamivudine. Initial enrollment was from December 2006 to October 2007 as previously described [13]. The additional inclusion criteria for this substudy were the patients who had continued with their initial antiretroviral regimens until 96 weeks. Any patient who had changed antiretroviral drug in the initial regimen due to any reason before 96 weeks was excluded. All enrolled patients were switched NRTI backbone from stavudine/lamivudine to tenofovir (Viread^®^)/lamivudine after 96-week treatment of both NNRTI-based regimens. They were monitored at time of switching (week 0), 12 and 24 weeks thereafter for serum phosphorus, serum magnesium, uric acid, creatinine, estimated glomerular filtration rate (eGFR), and urinalysis. The eGFR was calculated by the Modification in Diet in Renal Disease (MDRD) Study formula [14]. Changes in serum phosphorus was classified by grading system as follows: grade1, 2.5-2.7 mg/dL; grade 2, 2.0-2.4 mg/dL; grade 3, 1.0-1.9 mg/dL and grade 4, <1.0 mg/dL.

Analyses included all patients showing at least 1 visit after initiating tenofovir. Mean (± standard deviation, SD), median (interquartile range at 25^th ^and 75^th^, IQR) and frequencies (%) were used to describe patients' characteristic as appropriate. Chi-square test and Mann-Whitney U test were used to compare categorical and continuous variables between the two treatment groups, respectively. Wilcoxon Signed Ranks Test and pair-samples T test were used to compare measures between baseline and at 12 and 24 weeks after initiating tenofovir. The independent variables were evaluated with simple linear regression to identify the factors that were associated with low serum phosphorus level and low eGFR at week 24. By bivariate analysis, any independent variable with *P *value of less than 0.1 was included into the model of multiple regression analysis. The factors of age and serum phosphorus were examined as continuous variables and the remaining factors were examined as dichotomous variables. The regression coefficients (beta value) and its 95% confidence interval (CI) for each factor were computed. A positive regression weights for each factor means a one-point increase in factor results in an increase of beta value of mg/dL of serum phosphate. A negative weight has the opposite interpretation. Pearson's correlations were used to study the relationships between age and serum phosphorus. The Pearson's correlation coefficient (r) and coefficient of determination (r^2^) were computed. All analyses were performed using SPSS software version 15.0 (SPSS Inc., Chicago, IL, USA). A *P *value less than 0.05 was considered statistically significant. The study was reviewed and approved by the ethical review board of the Bamrasnaradura Infectious Diseases Institute and the Department of Disease Control, Ministry of Public Health.

## Results

**Figure 1:**
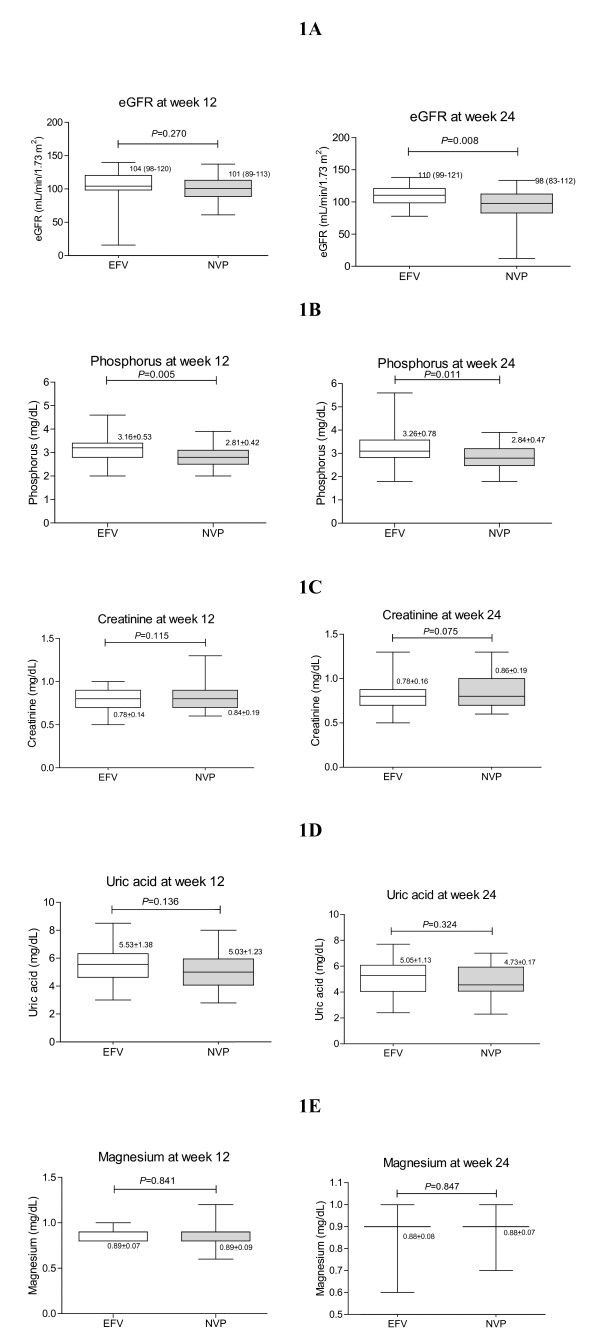
**Box plot of comparing mean ± SD and median (IQR) measures between the two groups at week 12 and 24**.

**Figure 2:**
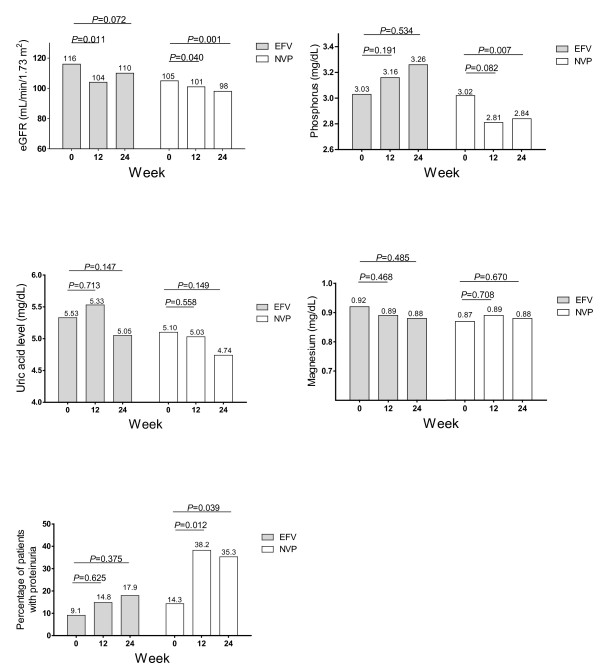
**Compare mean ± SD and median (IQR) measures between baseline (week 0) and subsequent weeks**.

A total of 62 patients met the inclusion criteria of this study and all followed until the end of this study. At week 0, all patients discontinued anti-tuberculous drugs. Of all, 28 patients were in the EFV group and 34 patients were in the NVP group. There were no significant differences in terms of demographic characteristics at week 0 as shown in table 1 (*P *> 0.05). Figure 1 compares measures between the two groups at week 12 and 24. For serum phosphorus between the EFV group vs. the NVP groups at week 12, proportion of patients who had grade I were 3 (11%) vs. 8 (24%) patients; and at week 24 were 2 (7%) vs. 6 (18%) patients, respectively. The proportion of those who had grade II serum phosphorus in the corresponding groups at week 12 were 2 (9%) vs. 7 (21%) patients; and at week 24 were 3 (11%) vs. 7 (21%) patients, respectively. The proportion of those who had grade III serum phosphorus at week 24 were 1 (4%) and 1 (3%) patients, respectively. Figure 2 compares means and median measures between baseline and subsequent weeks within each group. None of the patient developed acute renal failure and Fanconi syndrome during the follow-up period.

**Table 1:** Baseline characteristics at time of NRTI switching (week 0) between the two groups

Baseline characteristics	EFV group N = 28	NVP group N = 34	*P *value
Gender: Female	8 (29%)	13 (38%)	0.590

Age, years, mean ± SD	35.5 ± 6.9	38.7 ± 8.3	0.110

Body weight, kilograms, mean ± SD	62.3 ± 9.8	62.7 ± 11.0	0.888

CD4 count, cells/mm^3^, mean ± SD	342 ± 147	381 ± 154	0.307

Serum creatinine, mg/dL, mean ± SD	0.78 ± 0.22	0.76 ± 0.16	0.758

eGFR, mL/min, median (IQR)	116 (98-134)	105 (188-123)	0.195

Serum phosphorus, mg/dL, mean ± SD	3.0 ± 0.5	3.0 ± 0.7	0.952

Serum uric acid, mg/dL, mean ± SD	5.1 ± 1.3	5.3 ± 1.6	0.575

Serum magnesium, mg/dL, mean ± SD	0.87 ± 0.23	0.92 ± 0.20	0.391

Serum alkaline phosphatase, mg/dL, mean ± SD	87 ± 23	89 ± 24	0.743

**Figure 3:**
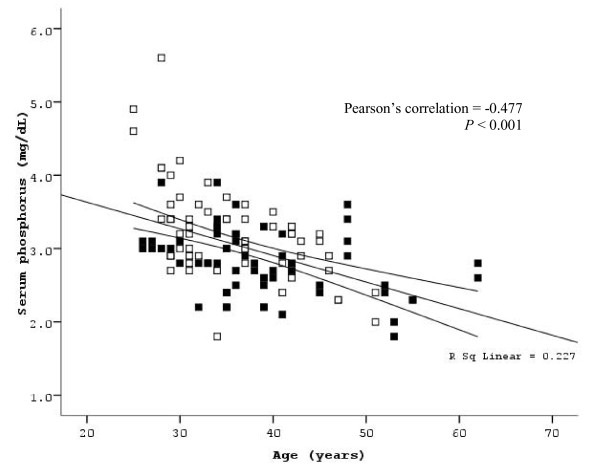
**Relationship between age of patients and combined serum phosphorus levels at week 12 and 24 after NRTI switching**. Lines represent regression prediction and 95 percent confidence intervals for the mean. Unfilled dot represents serum phosphorus in the EFV group; and filled dot represents serum phosphorus in the NVP group.

Table 2 and table 3 showed univariate and multivariate analysis of possible predicted factors that associated with low serum phosphorus and those associated with low eGFR at week 24 after NRTI switching. By multivariate analysis, three factors; included 'concurrent receiving nevirapine', 'old age' and 'low baseline serum phosphorus', were associated with low serum phosphorus level after 24 weeks of switching stavudine to tenofovir (*P *< 0.05). The factors 'concurrent receiving nevirapine' and 'low baseline eGFR' were associated with low eGFR at week 24 (*P *< 0.05). Relationship between age of patients and combined serum phosphorus levels at week 12 and 24 after NRTI switching is depicted in figure 3. The same trends were found at week 12 (*P *< 0.001, r = -0.540) and week 24 (*P *< 0.001, r = -0.434). At week 24, none of the patients experienced virological rebound or drug interruption.

**Table 2:** Univariate and multivariate analysis of possible factors associated with low serum phosphorus at week 24 after switching NRTI

Parameters	Univariate analysis			Multivariate analysis		
	
	Beta	95%CI of Beta	*P *value	Beta	95%CI of Beta	*P *value
Receiving efavirenz	0.320	0.099 to 0.739	0.011	0.321	0.098 to 0.714	0.011

Age	-0.434	-0.056 to -0.017	<0.001	-0.329	-0.049 to -0.006	0.015

Serum phosphorus at week 0	0.423	0.164 to 0.720	0.002	0.298	0.023 to 0.599	0.035

Negative HBsAg	-0.231	-1.278 to 0.054	0.071	0.070	-0.458 to 0.826	0.567

Female gender	0.237	-0.020 to 0.671	0.064	0.037	-0.306 to 0.405	0.781

**Table 3:** Univariate and multivariate analysis of possible factors associated with low eGFR at week 24 after switching NRTI

Parameters	Univariate analysis			Multivariate analysis		
	
	Beta	95%CI of Beta	*P *value	Beta	95%CI of Beta	*P *value
eGFR at week 0	0.572	0.342 to 0.743	<0.001	0.529	0.296 to 0.707	<0.001

Receiving efavirenz	0.331	3.734 to 24.596	0.009	0.245	1.438 to 19.521	0.024

Age	-0.228	-1.325 to 0.063	0.074	-0.016	-0.649 to 0.562	0.886

## Discussion

Despite tenofovir-containing NRTI backbone regimen is effective and well tolerated, the potential for renal toxicity still exists, especially in the patients with vulnerable kidney conditions [5,15,16]. The previous reviews showed that mild tubular impairment is found in a substantial proportion of patients who treated with tenofovir and tends to increase with cumulative exposure [17-21]. However, the onset of tenofovir-associated renal toxicity occurred widely after receiving tenofovir [22]. The present study reveals significant decreases in eGFR within the first three months after the patients were switched from stavudine to tenofovir. Furthermore, decrements of eGFR progressed over time under tenofovir exposure together with persistent hypophosphatemia, lower serum uric acid level, and higher proportion of patients with proteinuria, especially in the patients receiving nevirapine-based ART. This is explained by tenofovir itself primarily involves in renal tubular dysfunction and it may lead to Fanconi syndrome with or without renal impairment. It less frequently effect on glomerular abnormalities. This renal proximal tubular dysfunction is manifested by decreased tubular reabsorption of phosphate resulting in hypophosphatemia. Although hypophosphatemia is considerably common in HIV-infected patients, other secondary causes of increase in urinary loss in these stable patients with normal renal function are unlikely.

Tenofovir-associated renal dysfunction can occur as a result of complex drug-drug interactions among antiretroviral drugs [22]. Most previous reports demonstrated that this event have developed in HIV-infected patients receiving a regimen containing ritonavir-boosted protease inhibitors or didanosine [22,23]. Interestingly, we found that concomitant administration of tenofovir with two different non-nucleoside reverse transcriptase inhibitors appeared to have considerably different effects on renal toxicity. Those aforementioned findings were almost not recognized in the patients concurrently receiving tenofovir in an efavirenz-based ART. Although nevirapine is extensively metabolized via cytochrome P450 metabolism to several hydroxylated metabolites, other isozymes may be involved with its metabolism [24]. In a previous pharmacokinetic study, approximately 81% of a radiolabeled dose was recovered in the urine, with greater than 80% of that made up of glucuronide conjugates of hydroxylated metabolites, and less than 3% by unchanged drug [25]. On the other hand, tenofovir disoproxil fumarate is the prodrug of the active ingredient tenofovir. It is neither a substrate nor an inhibitor of cytochrome enzymes, therefore low potential for tenofovir-nevirapine interaction via the cytochrome systems [15,16]. Tenofovir disoproxil fumarate is metabolized by diester hydrolysis to tenofovir, which is then metabolized by phosphorylation to the pharmacologically-active metabolite tenofovir diphosphate. This drug is principally secreted into the urine via multidrug resistance protein (MRP) at proximal cells of renal tubule [26]. Given that a majority of metabolite compounds of both nevirapine and tenofovir are eliminated via kidney, it might be hypothesized that potential drug-drug interactions may occur at this site. A recent study in animal model treated with tenofovir revealed increased number and irregular shape of mitochondria with sparse fragmented cristae in renal proximal tubules. Interfering the elimination of tenofovir may result in its accumulation and lead to toxicity. Therefore, further studies regarding patho-physiology of the renal impairment in tenofovir-containing backbone NRTI plus nevirapine on this aspect are warranted. In addition, a pharmacogenetic study revealed that polymorphisms in the ABCC2 gene encoding for the MRP2 was associated with proximal renal tubular dysfunction in patients receiving tenofovir [27]. Thus, host-genetic predisposition may play role.

Interestingly, significantly hypophosphatemia occurred in the patients who concurrently received tenofovir; however, the clinical significance of these changes is not well understood. The evidence from this study showed that overall eGFR is substantially declined with accumulative tenofovir exposure although no patient discontinued the study due to renal adverse events and there were no cases of Fanconi syndrome. An incomplete reversibility of tenofovir-related renal toxicity, by assessing eGFR, in HIV-infected men had been observed in a recent study [28]. Over the past several years, stavudine has been recommended as part of a preferred NRTI backbone in combined with nevirapine in the resource-constrained countries [10]. On the other hand, nevirapine-based ART is still a key regimen to scale up treatment of HIV in such countries [10]. Phasing out of stavudine by replaced it with tenofovir is undertaken; therefore the policy of close monitoring of tenofovir-associated renal toxicity for the safety in this strategy is required. The safety issue will be very important in many resource-constrained setting, where laboratory monitoring is less accessible.

Identification and reversal of potentially modifiable risk factors prior to drug use is beneficial to lower the incidence of renal injury. As we have known that multiple factors influenced in the declines in renal function in our HIV-infected patients. The result presented here shows that age is another factor which was associated with hypophosphatemia; however, it was not a predictor of declining in eGFR. The other previous reported risk factors associated with renal toxicity included the concurrent use of other nephrotoxic medications, the use of nonsteroidal anti-inflammatory drugs, the use of a protease inhibitor, as well as co-morbidities, such as hypertension and diabetes [15,16].

There are a number of limitations need to be addressed in the present study. First, this study is not primarily designed to assess the tenofovir-related adverse events and association between either serum phosphorus or eGFR and other parameters. Thus, the other potential factors might not be well-controlled. Second, our sample size is relatively small and the follow-up period is relatively short. Our findings should be confirmed by a larger scale of long-term cohort and prospective randomize trial. Nonetheless, the findings revealed a tendency of an association between receiving tenofovir-containing nevirapine-based ART and poor renal outcomes at week 12 and 24 after switching stavudine to tenofovir. However, this is the first clinical trial that has shown this relationship so far. Third, the evidence of urinary loss of phosphorus was not definitely confirmed, such as fractional excretion of phosphorus. As mentioned earlier, other secondary reason to explain the persistent hypophosphatemia after a short-period of tenofovir introduction in these stable patients are difficult. Ultimately, the differences in demographics and genetics may play role on the frequency of these toxicities. All enrolled patients in the study were Thais; therefore, this may not be applicable to other ethnics.

In summary, the present study provides promising clinical data in terms of renal impairment progresses over time under a short period of tenofovir exposure. Moreover, the frequency of tenofovir-associated renal impairment was significantly higher in HIV-infected patients receiving tenofovir/lamivudine/nevirapine compared to tenofovir/lamivudine/efavirenz and the progress of renal impairment in this scenario is multifactorial. Although tenofovir plus emtricitabine or lamivudine is a preferred NRTI backbone regimen, close monitoring of renal function by measuring creatinine clearance and serum phosphorus is recommended, particularly in the settings where laboratory monitoring is less accessible. This finding should be validated in a larger scale of study and further studies regarding patho-physiology of the renal impairment in tenofovir/lamivudine/nevirapine needs to be explored.

## Competing interests

The authors declare that they have no competing interests.

## Authors' contributions

WM participated in the design of the study, statistical analysis and draft the manuscript. WM, AL, WP, PT, BS, AN, SN, and SS participated in the design of the study and draft the manuscript. All authors read and approved the final manuscript.
